# Cervical cancer screening aided by artificial intelligence, China

**DOI:** 10.2471/BLT.22.289061

**Published:** 2023-05-01

**Authors:** Xingce Zhu, Qiang Yao, Wei Dai, Lu Ji, Yifan Yao, Baochuan Pang, Bojana Turic, Lan Yao, Zhiyong Liu

**Affiliations:** a School of Medicine and Health Management, Tongji Medical College, Huazhong University of Science and Technology, Wuhan, 430030 China.; b School of Political Science and Administration, Wuhan University, Wuhan, China.; c Landing Artificial Intelligence Center for Pathological Diagnosis, Wuhan University, Wuhan, China.; d Landing Artificial Intelligence Industry Research Institute, Wuhan, China.

## Abstract

**Objective:**

To implement and evaluate a large-scale online cervical cancer screening programme in Hubei Province, China, supported by artificial intelligence and delivered by trained health workers.

**Methods:**

The screening programme, which started in 2017, used four types of health worker: sampling health workers, slide preparation technicians, diagnostic health workers and cytopathologists. Sampling health workers took samples from the women on site; slide preparation technicians prepared slides for liquid-based cytology; diagnostic health workers identified negative samples and classified positive samples based on the Bethesda System after cytological assessment using online artificial intelligence; and cytopathologists reviewed positive samples and signed reports of the results online. The programme used fully automated scanners, online artificial intelligence, an online screening management platform, and mobile telephone devices to provide screening services. We evaluated the sustainability, performance and cost of the programme.

**Results:**

From 2017 to 2021, 1 518 972 women in 16 cities in Hubei Province participated in the programme, of whom 1 474 788 (97.09%) had valid samples for the screening. Of the 86 648 women whose samples were positive, 30 486 required a biopsy but only 19 495 had one. The biopsy showed that 2785 women had precancerous lesions and 191 had invasive cancers. The cost of screening was 6.31 United States dollars (US$) per woman for the public payer: US$ 1.03 administrative costs and US$ 5.28 online screening costs.

**Conclusion:**

Cervical cancer screening using artificial intelligence in Hubei Province provided a low-cost, accessible and effective service, which will contribute to achieving universal cervical cancer screening coverage in China.

## Résumé

**Objectif:**

Déployer et évaluer un programme en ligne de grande envergure destiné au dépistage du cancer du col de l'utérus dans la province du Hubei, en Chine, reposant sur l'intelligence artificielle et mis en œuvre par des agents de santé formés à cet effet.

**Méthodes:**

Le programme de dépistage, qui a débuté en 2017, s'appuyait sur quatre types d'agents de santé: des agents chargés de prélever les échantillons, des techniciens chargés de préparer les lames, des agents chargés d'établir le diagnostic et des cytopathologistes. Les premiers ont prélevé des échantillons auprès des femmes sur place, les deuxièmes ont préparé les lames pour la cytologie en phase liquide, les troisièmes ont identifié les échantillons négatifs et classé les échantillons positifs selon le système de Bethesda après analyse cytologique à l'aide d'une intelligence artificielle en ligne, tandis que les cytopathologistes ont examiné les échantillons positifs et signé les rapports de résultats en ligne. Le programme était constitué de scanners entièrement automatisés, d'une intelligence artificielle en ligne, d'une plateforme de gestion du dépistage en ligne, ainsi que d'appareils de téléphonie mobile pour les services de dépistage. Nous avons évalué la viabilité, les performances et le coût d'un tel programme.

**Résultats:**

Entre 2017 et 2021, 1 518 972 femmes résidant dans 16 villes de la province du Hubei ont participé au programme, et 1 474 788 (97,09%) d'entre elles ont présenté des échantillons valides pour le dépistage. Sur les 86 648 femmes dont les échantillons se sont révélés positifs, 30 486 ont eu besoin d'une biopsie, mais seulement 19 495 en ont effectué une. La biopsie a montré que 2785 femmes présentaient des lésions précancéreuses et que 191 étaient atteintes de cancers invasifs. Le coût du dépistage s'élevait à 6,31 dollars américains (USD) par femme pour le payeur public: 1,03 USD pour les frais administratifs et 5,28 USD pour le dépistage en ligne.

**Conclusion:**

Le dépistage du cancer du col de l'utérus assisté par intelligence artificielle dans la province du Hubei est un service abordable, accessible et efficace, qui contribuera à garantir une couverture universelle en la matière dans le pays.

## Resumen

**Objetivo:**

Implementar y evaluar un programa de detección del cáncer del cuello uterino en línea a gran escala en la provincia de Hubei (China), con el apoyo de la inteligencia artificial y a cargo de trabajadores sanitarios formados.

**Métodos:**

El programa de detección, que comenzó en 2017, utilizó cuatro tipos de trabajadores sanitarios: trabajadores sanitarios de muestreo, técnicos de preparación de portaobjetos, trabajadores sanitarios de diagnóstico y citopatólogos. Los trabajadores sanitarios de muestreo tomaban muestras de las mujeres in situ, los técnicos de preparación de portaobjetos preparaban los portaobjetos para la citología mediante líquidos, los trabajadores sanitarios de diagnóstico identificaban las muestras negativas y clasificaban las muestras positivas según el Sistema Bethesda tras la evaluación citológica mediante inteligencia artificial en línea, y los citopatólogos revisaban las muestras positivas y firmaban los informes de los resultados en línea. El programa utilizó escáneres totalmente automatizados, inteligencia artificial en línea, una plataforma de gestión de la detección en línea y dispositivos de telefonía móvil para proporcionar servicios de detección. Se evaluaron la sostenibilidad, el rendimiento y el coste del programa.

**Resultados:**

Entre 2017 y 2021, 1 518 972 mujeres de 16 ciudades de la provincia de Hubei participaron en el programa, de las que 1 474 788 (97,09%) tenían muestras válidas para la detección. De las 86 648 mujeres cuyas muestras dieron positivo, 30 486 necesitaron una biopsia, pero solo 19 495 se sometieron a ella. La biopsia mostró que 2785 mujeres tenían lesiones precancerosas y 191 cánceres invasivos. El coste de la detección fue de 6,31 dólares estadounidenses (US$) por mujer para el organismo público pagador: US$ 1,03 de costes administrativos y US$ 5,28 de costes de detección en línea.

**Conclusión:**

La detección del cáncer del cuello uterino mediante inteligencia artificial en la provincia de Hubei proporcionó un servicio de bajo coste, accesible y eficaz, que contribuirá a lograr la cobertura universal de detección del cáncer del cuello uterino en China.

## ملخص

**الغرض:**

تنفيذ وتقييم برنامج واسع النطاق لفحص سرطان عنق الرحم عبر الإنترنت في مقاطعة هيوبي في الصين، بدعم من الذكاء الاصطناعي، وتقديمه بواسطة العاملين تحت التدريب في المجال الصحي.

**الطريقة:**

استخدم برنامج الفحص، الذي بدأ في عام 2017، أربعة أنواع من العاملين في المجال الصحي: العاملين في جمع العينات، وفنيي إعداد الشرائح، والعاملين في التشخيص، وأخصائيي الأمراض الخلوية. قام العاملون في العينات بجمع عينات من النساء في الموقع، وقام فنيو إعداد الشرائح بإعداد شرائح لعلم الخلية القائم على السوائل، وقام العاملون في التشخيص بتحديد العينات السلبية، والعينات الإيجابية المصنفة بناءً على نظام Bethesda بعد التقييم الخلوي باستخدام الذكاء الاصطناعي عبر الإنترنت، وقام علماء الأمراض الخلوية باستعراض العينات الإيجابية، وتوقيع تقارير النتائج على الإنترنت. استخدم البرنامج الماسحات الضوئية المؤتمتة بالكامل، والذكاء الاصطناعي عبر الإنترنت، ومنصة إدارة الفحص عبر الإنترنت، وأجهزة الهاتف المحمول لتقديم خدمات الفحص. وقمنا بتقييم استدامة، وأداء، وتكلفة البرنامج.

**النتائج:**

في الفترة من 2017 إلى 2021، شاركت 1518972 امرأة في 16 مدينة في مقاطعة هيوبي في البرنامج، من بينهن 1474788 (%97.09) كانت لديهن عينات صالحة للفحص. من بين 86648 امرأة كانت عيناتهن إيجابية، احتاجت 30486 منهم إلى خزعة، ولكن 19495 فقط كانت لديهن عينة. أظهرت الخزعة أن 2785 امرأة مصابات بأضرار سرطانية محتملة، و191 مصابات بحالات سرطان متدهورة. بلغت تكلفة الفحص 6.31 دولارًا أمريكيًا ($) لكل امرأة كمبلغ عام: 1.03 دولار أمريكي تكاليف إدارية، و5.28 دولارات أمريكية تكاليف للفحص عبر الإنترنت.

**الاستنتاج:**

إن فحص سرطان عنق الرحم باستخدام الذكاء الاصطناعي في مقاطعة هيوبي، قدم خدمة منخفضة التكلفة، وسهلة الوصول، وفعالة، مما سيسهم في تحقيق تغطية شاملة لفحص سرطان عنق الرحم في الصين.

## 摘要

**目的:**

在中国湖北省，接受过培训的卫生工作者在人工智能技术的支持下实施并评估了一项大规模的在线宫颈癌筛查项目。

**方法:**

该筛查项目自 2017 年起开始启用，需四种卫生工作者协同工作：采样卫生工作者、玻片制备技术人员、诊断卫生工作者和细胞病理学家。采样卫生工作者从现场女性身上采集样本；玻片制备技术人员制备液基细胞学载玻片；诊断卫生工作者在使用在线人工智能软件进行细胞学评估后，根据贝塞斯达系统 (Bethesda System) 识别阴性样本并分拣出阳性样本；细胞病理学家仔细研究阳性样本，并在线签署结果报告。该项目使用全自动扫描仪、在线人工智能软件、在线筛查管理平台和移动电话设备来提供筛查服务。我们评估了该项目的可持续性、成效和成本。

**结果:**

在 2017 年至 2021 年期间，湖北省 16 个城市的 1,518,972 名女性参加了该项目，且其中 1,474,788 名女性（占 97.09%）提供了有效筛查样本。在样本呈阳性的 86,648 名女性中，30,486 名需进行活检，但仅 19,495 名接受了此检查。活检结果显示，2,785 名女性存在癌前病变现象，且 191 名女性已患有浸润性癌症。公共付款机构承担的筛查费用为每位女性 6.31 美元（US$）：1.03 美元管理费用和 5.28 美元在线筛查费用。

**结论:**

湖北省利用人工智能技术进行宫颈癌筛查打造了一种既具有低成本又方便有效的服务，将有助于在中国普遍推行宫颈癌症筛查。

## Резюме

**Цель:**

Реализовать и оценить широкомасштабную онлайн-программу скринингового обследования рака шейки матки с использованием искусственного интеллекта и привлечением обученных сотрудников сферы здравоохранения в провинции Хубэй, Китай.

**Методы:**

В начавшейся в 2017 году программе скринингового обследования участвовали четыре типа сотрудников сферы здравоохранения: медицинские работники, осуществляющие отбор образцов, техники, занимающиеся приготовлением микроскопических препаратов, диагносты и цитологи. Медицинские работники, осуществляющие отбор образцов, брали образцы у женщин в исследовательских центрах; техники, занимающиеся приготовлением микроскопических препаратов, готовили микроскопические препараты для проведения жидкостной цитологии; диагносты определяли отрицательные образцы и классифицировали положительные образцы на основе классификации Бетесда после цитологической оценки с использованием искусственного интеллекта в режиме онлайн; цитологи изучали положительные образцы и подписывали отчеты о результатах в режиме онлайн. В программе использовались: полностью автоматизированные сканеры, онлайн-искусственный интеллект, онлайн-платформа управления скрининговым обследованием и мобильные телефонные устройства для предоставления услуг скрининга. Проводилась оценка устойчивости, эффективности и стоимости программы.

**Результаты:**

С 2017 по 2021 год в программе приняли участие 1 518 972 женщины в 16 городах провинции Хубэй, из которых у 1 474 788 (97,09%) имелись достоверные образцы для скринингового обследования. Из 86 648 женщин, чьи образцы оказались положительными, 30 486 потребовалась биопсия, но провели ее только 19 495. По результатам биопсии у 2785 женщин были обнаружены предопухолевые состояния, а у 191 – инвазивный рак. Стоимость скринингового обследования составляла 6,31 долл. США (US$) на одну женщину для юридического лица: 1,03 долл. США на административные расходы и 5,28 долл. США на скрининговое обследование в режиме онлайн.

**Вывод:**

Скрининговое обследование рака шейки матки с использованием искусственного интеллекта в провинции Хубэй предоставил недорогую, доступную и эффективную услугу, которая будет способствовать достижению всеобщего охвата населения услугами скрининга рака шейки матки в Китае.

## Introduction

Cervical cancer is a threat to women’s health. According to data released by the International Agency for Research on Cancer, the cancer research agency of the World Health Organization (WHO), 0.6 million women worldwide were diagnosed with cervical cancer and 0.3 million died from cervical cancer in 2020.^1^ These numbers represent 3.1% of all newly diagnosed cancers worldwide (making cervical cancer the ninth leading cancer diagnosis), and 7.7% of global female cancer deaths (making this cancer the fourth leading cause of death in women).^1^ WHO released the *Global strategy to accelerate the elimination of cervical cancer* in 2020, with the goal of eliminating cervical cancer (a rate of fewer than 4 cases per 100 000 women) by 2030. One of the targets is that 70% of women are screened with a high-performance test by 35 years of age, and again by 45 years of age. Thus far, 194 countries, including China, have made the commitment to eliminate cervical cancer.^2^^,^^3^

In contrast to developed countries, where the incidence of cervical cancer is low and prevention is the primary focus, low- and middle-income countries, such as China, are still far from achieving this goal,^4^ and many women are still not screened or are under-screened.^5^^–^^10^ However, the costs of deoxyribonucleic acid (DNA) detection of human papillomavirus in large populations and the expansion of the cervical cancer vaccine, which are the main cervical cancer prevention measures in developed countries,^11^ are high. Therefore, the use of relatively inexpensive cytology methods with good sensitivity and specificity is needed to achieve universal and reliable screening coverage in large populations in low- and middle-income countries. WHO has also suggested that cytological techniques still play an important role in screening and triage.^12^^,^^13^ However, the widespread use of cytology in China is restricted by the lack of trained cytopathologists and dedicated laboratories.^14^^,^^15^ Furthermore, differences in proficiency of cytopathologists and the difficulty in maintaining diagnostic consistency among the limited number of cytopathologists lead to inconsistencies in the quality of diagnosis.^14^ Resolving all these problems is important to facilitate the use of cytology in screening.

The need for improved digital health in remote and deprived areas of China has recently been highlighted.^16^^–^^18^ The National Health Commission is encouraging the incorporation of emerging artificial intelligence and internet technologies in screening to enhance cervical cancer prevention and treatment.^19^ As machine learning develops, artificial intelligence has advantages in cervical cytology^14^^,^^20^^,^^21^ and colposcopy,^22^^–^^24^ and will gradually outperform human clinicians in performing these procedures.^25^^–^^27^ WHO has also suggested that artificial intelligence can advance the screening process.^13^ Preparation and interpretation of cervical cell slides using artificial intelligence can replace or assist cytopathologists, which addresses the problem of a lack of experienced medical personnel in screening.^14^^,^^20^^,^^21^^,^^26^^–^^28^ In addition, improved access to health-care services in remote areas and lower health-care costs are both made possible by the internet, cloud computing and mobile devices.^14^^,^^20^

Based on the above-mentioned feasibility studies and policies, we implemented an online cervical cancer screening programme aided by artificial intelligence in Hubei Province, China. This programme aimed to: increase access to relevant health services for women in underdeveloped areas who have limited screening coverage; alleviate some of the health inequities these women face; and contribute to the achievement of universal cervical cancer screening coverage and cervical cancer elimination goals in China. In this paper, we describe this technology-based service-delivery model and evaluate its success in terms of: programme sustainability (number of women screened annually in different cities); performance (number of women with confirmed diagnoses and some important indicators of screening outcomes, such as number of inadequate and positive slides); and costs for the technology provider and public payer. We also discuss the challenges encountered in the implementation of this programme and the strategies developed to overcome these issues.

## Methods

### Study setting and preparation

We first implemented our screening programme among rural women of low socioeconomic status and gradually expanded it to urban women in Hubei Province, China, from 2017 to 2021. Hubei Province has 36.32 million urban residents and 21.43 million rural residents, and 12.67 million women aged 35 to 64 years are eligible for cervical cancer screening.^29^ In the screening, all women are initially evaluated using cytology, and women with abnormal cytology results are referred for colposcopy-directed biopsies, if necessary.

This screening programme was established through an open-tender process to select a screening contractor and technology provider. The Landing Artificial Intelligence Industry Research Institute in Wuhan (hereafter called Landing), which is certified to perform cytological and histopathological diagnosis and has an online cytological assessment system using artificial intelligence, won the tender in 2017. Screening can be performed in areas where mobile telephones can receive fourth-generation (4G) mobile communication technology signals, which allow screening data to be sent through the network. By the end of 2016, all administrative villages and urban households in Hubei Province had full 4G network coverage.

Before implementation of the programme, we conducted a situational analysis of the routes to screening services and the referral routes for treatment for women with positive cytology results. Landing, local health centres, and maternal and child health hospitals were the main screening service providers. Local health centres, which may have a gynaecology department, are distributed in villages and urban communities and provide primary health care. Local maternal and child hospitals, generally one for each county and city, are secondary or tertiary hospitals dedicated to providing comprehensive health care to all women, infants and children in the region.

We also established partnerships with local health commissions and women’s federations. These groups and the local health centres in cities across Hubei Province typically conduct screening mobilization in rural and urban communities each year between January and March. Through on-site meetings and online announcements, women were informed about cervical health, the importance of early prevention and screening, the time and place of screening, the factors to consider before screening (for example, ideally not to screen during menstruation) and the tests offered to women in the screening. Women also received short messages on their mobile telephones reminding them to participate in the screening. Around September each year, women in villages and communities with insufficient participation in the cytology-based screening, as determined by aggregated screening data, were mobilized once more.

### Health workers

We assigned four types of trained health workers to conduct the cytology-based screening: sampling health workers; slide preparation technicians; diagnostic health workers; and cytopathologists (Box 1). These health workers mostly came from local health centres, maternal and child hospitals, and Landing, and were their regular medical staff. Landing undertook the training of the health workers in the use of the screening system.

Box 1 **Institutions and staff involved in the online cervical cancer screening programme, Hubei Province, China, January 2017–December 2021**

***Rural and urban health centres, and local maternal and child health hospitals***

Sampling health workers

- Main tasks: collect identity information and cervical exfoliated cells from the women.

***Landing Artificial Intelligence Industry Research Institute***

Slide preparation technicians

- Main tasks: prepare slides for diagnosis.

Diagnostic health workers

- Main tasks: Identify negative cases and carry out simple classification of positive cases based on the Bethesda System after cytological assessments by the online artificial intelligence system.

Cytopathologists

- Main tasks: Review all positive cases and 10% of negative cases (randomly selected) classified by diagnostic health workers, and sign reports of the results.

The sampling health workers from rural and urban health centres were assigned to collect and fix cervical exfoliated cells for the women being screened. Local maternal and child health hospitals in urban areas provided urban women with access to sampling. Given that some trained sampling health workers in local health centres may lack proficiency and experience in sampling, particularly in rural health centres, the local maternal and child health hospital can send someone to help with the sampling process. Slide preparation technicians used specific devices for slide coding and slide preparation for liquid-based cytology. The trained diagnostic health workers were assigned to identify negative cases, and classify the cases with abnormalities based on the Bethesda System after the cytological assessment using online artificial intelligence had been done. Trained cytopathologists then reviewed the positive cases and their classification, and checked a random 10% of cases classified as negative by diagnostic health workers for quality control.

The sampling health workers were employed full-time at local health centres or maternal and child health hospitals, where they oversaw the provision of daily health-care services to residents of a specific area; sampling women in the area was a regular part of their work. The salaries of these health workers were paid by different medical institutions. Landing employed the diagnostic health workers, slide preparation technicians and some of the trained cytopathologists on a full-time basis. Other cytopathologists were from large hospitals, such as Tongji Hospital and Union Hospital in Wuhan. These cytopathologists were paid by Landing based on the number of cases they diagnosed online in their spare time; these cytopathologists undertook this work to supplement their income.

All of the health workers had previously received medical education training, but no formal training in online cytological assessment using artificial intelligence. The sampling health workers attended a 1-day screening meeting and training on sampling standards. Slide preparation technicians were proficient in liquid-based cytology slide preparation before joining Landing, and required no additional training after joining the screening programme. Senior pathologists employed by Landing provided all diagnostic health workers with 2–3 weeks of specialized training on digital cytological assessment, including: grading criteria of the Bethesda System and the Papanicolaou test; procedures for differentiating between negative and positive cases by reviewing digital cell images online; and the online operational workflow. The cytopathologists did not require medical skills training, but had 3–5 days of training on the operation of online cytological assessment and issuing of reports. Courses for the diagnostic health workers were held at the Landing facilities; other training was held at the workplace of the health workers. All the health workers were supervised through an online screening management platform (Landing, Wuhan, China), as the data generated at each step of their operation was recorded.

### Implementation

**Fig. 1:**
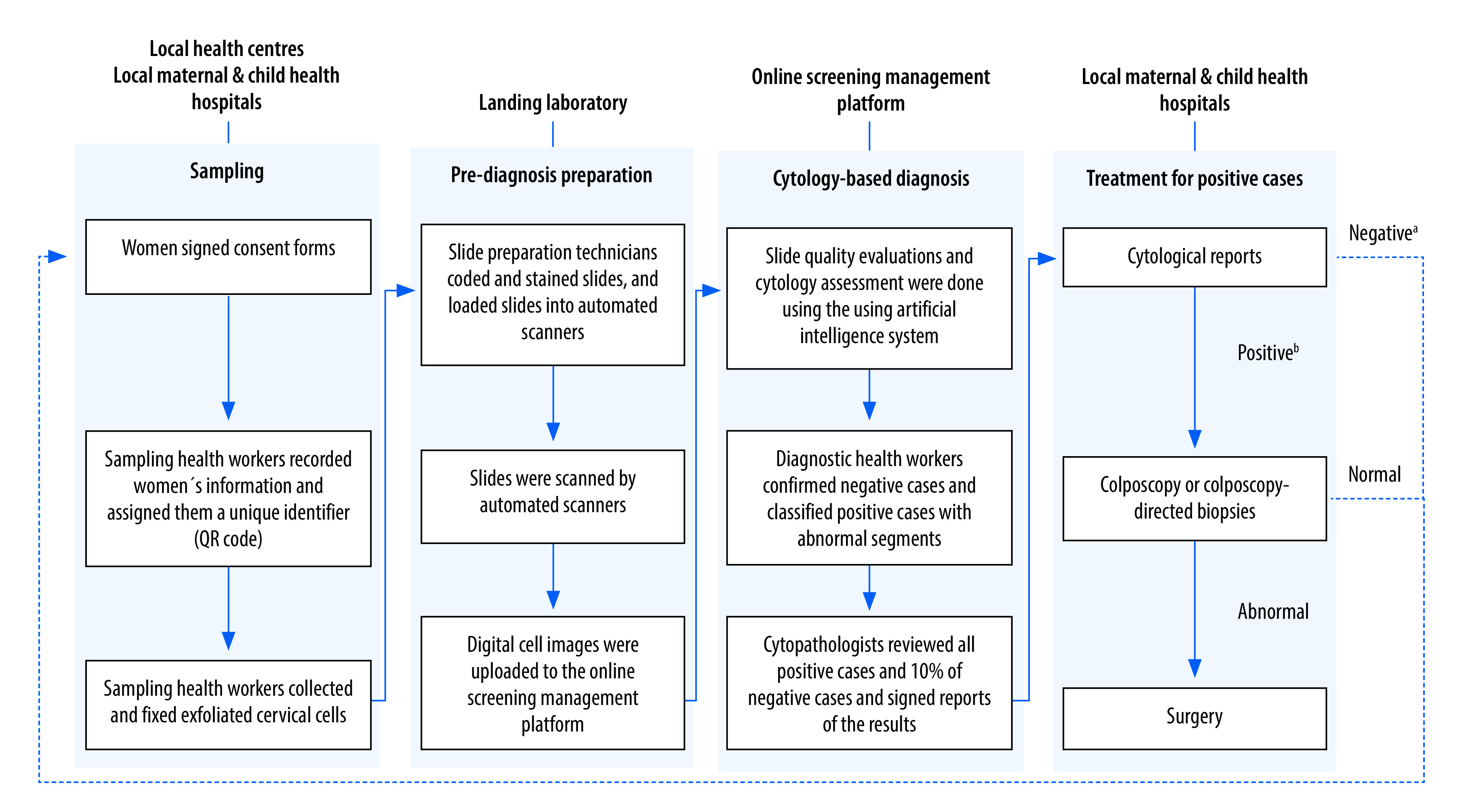
Flowchart of processes in the online cervical cancer screening programme, by phase, Hubei Province, China, January 2017–December 2021

The implementation of the screening programme had four phases: sampling, prediagnostic preparation, cytology-based assessment and treatment for positive cases (Fig. 1).

QR: quick response.

^a^ These electronic reports could be accessed by the women online.

^b^ These paper reports needed to be picked up by the women at a designated health centre.

Each city in Hubei Province developed its own screening workplan. In accordance with this workplan, the sampling health workers took samples from women who signed consent forms at local health centres or maternal and child health hospitals over the screening period, usually 1 week or more. First, they used a networked identity card reader to capture women’s identity information and archive it in the online screening management platform. Each woman was assigned a unique quick response (QR) code that could be scanned with a mobile device, such as a smartphone or tablet, to access her online cytological assessment report. Next, the sampling health workers collected and fixed the exfoliated cervical cells and arranged for their transport to Landing’s Wuhan laboratory. For women in villages or communities who had difficulty travelling to the centres, sampling health workers were taken by bus to provide a door-to-door sampling service.

After specimens from the sampling facilities arrived at the laboratory, the slide preparation technicians prepared slides for liquid-based cytology. Then, the slides were loaded into fully automated scanners (Landing, Wuhan, China) for scanning and digital cell image uploading. The QR code on each slide was used to match it with the woman recorded in the system. In some cities, a sub-laboratory was established with the help of Landing, which enabled slide preparation and data uploading locally. Furthermore, a portable hand-held automated scanner (Landing, Wuhan, China) could be sent to remote villages or communities so that data could be uploaded to the online screening management platform immediately after sampling. This strategy was used to overcome the difficulty and high cost of preserving and transporting specimens in remote villages or communities with few women.

After scanning the samples, the online artificial intelligence system evaluated the adequacy of the slides and made an initial cytological assessment. Women whose samples were considered inadequate needed to be resampled; samples that were adequate were classified as negative (negative for intraepithelial lesion or malignancy) or positive. All samples read by the online artificial intelligence system were reviewed by the diagnostic health workers. These health workers logged into the screening management platform to identify negative cases and classify positive samples based on the Bethesda System.

The main task of the cytopathologists was to confirm or adjust the classification of samples with abnormalities, and to sign the reports of the results on the screening management platform. For quality control, the cytopathologists also reviewed 10% of the negative samples (randomly selected) that had been classified by the diagnostic health workers. The report was available between 1 and 10 days after the specimen arrived at the Landing laboratories. Women with a negative diagnosis could directly view their report online using WeChat (Tencent, Guangzhou, China) on their mobile telephone devices and they were placed on a list for the next cycle of the screening programme.

Women with positive samples could not view their report online; instead, they were sent a message informing them of a possible abnormal diagnosis and the place where they could obtain their paper report. Landing sent the paper reports of cytological abnormalities to the local health centre, or maternal and child health hospital in the area where the women with positive samples lived. When the women picked up their reports, they were told where they could obtain a free re-examination – usually at the local maternal and child hospital. Women were classified as requiring a colposcopy-directed biopsy if their physicians recommended a biopsy during re-examination.

We calculated the cost of the screening programme from two perspectives: the public payer (provincial government finance department) and the technology provider (Landing).

### Technology

The technology and devices we used for screening were able to meet our screening demands. A fully automated scanner in the laboratory can load 200 slides at a time, and automatically change slides in sequence to scan and upload data to the online screening management platform. Each slide took fewer than 2 minutes to be scanned by the scanner and the data uploaded to the online system for cytological assessment using the online artificial intelligence system, and a single scanner could process 1000 slides a day. With 50 networked automated scanners, the Landing laboratory was able to perform around 30 000 high-throughput cytological assessments a day with an average diagnostic time of 1.5 minutes per sample, which is equivalent to the maximum workload of a pathologist for a year with quality assurance and error avoidance.^25^^,^^30^ A portable hand-held scanner can only load one or two slides at a time, but it scans and uploads with the same performance as the laboratory scanner. In addition, the diagnostic output within a certain time could be increased by adding more scanners or opening a sub-laboratory elsewhere with these networked devices.

The accuracy of the online cytological assessment system aided by artificial intelligence has been improving as cell images of screened women have gradually accumulated in the digital cell image library. Landing ran well-defined cell data through the system at many different times to improve the accuracy of the identification of slides as negative, positive or inadequate.^26^^,^^27^ On the basis of the Bethesda System, negative meant negative for intraepithelial lesion or malignancy, and the rest of the grades were positive. Inadequate slides had fewer than 5000 epithelial cells in the field of view, a large accumulation of cells or a blurred background, typically because of irregular sampling procedures. Adequate slides generated high-definition microelectronic cell images, and were given a comprehensive score based on over 1000 indicators analysed by the online artificial intelligence system. The 20 most likely abnormal segments were selected for diagnostic health workers and cytopathologists to review.

The online screening management platform, which was built on AliCloud (Alibaba, Hangzhou, China), synchronized screening results between the network and mobile device software. China’s Ministry of Public Security has certified this platform as a National Information Security Classified Protection Level 3. This rating is the highest level of national certification for the information security management capabilities of non-banking institutions. The diagnostic health workers and cytopathologists used the account and password given by Landing to log into the platform and review the slides. Only the case number, age, current assessment result and a panoramic view of the cell images were displayed during the review. Information that could be used to identify an individual was encrypted. The diagnostic health workers and cytopathologists completed their online reviews on their work computers or mobile devices (smartphones, tablets, laptops or personal computers) without compromising confidentiality because of the protection within the system and professional ethical conduct.

### Data extraction

With the assistance of Landing, we extracted data from the online platform into an Excel (Microsoft, Redmond, USA) spreadsheet for data integration and analysis. The data we obtained and analysed were big data and any information on the women’s identities was removed. Information about patient safety and confidentiality was the responsibility of the medical service providers. We also used Excel to record and quantify some information management indicators for the screening programme.

### Ethical considerations

Before samples were taken, women signed a written informed consent form which noted that their samples could be used in big data studies. The data we obtained for this study did not include any sensitive information such as women’s identity or human tissue.

## Results

A total of 1 518 972 eligible women who signed consent forms participated in the programme in 16 cities of Hubei Province between 2017 and 2021 (Table 1). Of these women, 44 184 (2.91%) had inadequate samples and could not be classified, 1 388 140 (91.39%) had no abnormalities on cytological assessment and 86 648 (5.70%) were classified as positive (Table 2). Of the women with positive diagnosis, 30 486 were recommended to have a colposcopy-directed biopsy but only 19 495 (63.95%) had the procedure (Table 2). Of the women who had a biopsy, 2785 were diagnosed with cervical precancerous lesions and 191 with infiltrating cervical cancer (Table 2).

**Table 1:** Number of women screened for cervical cancer through the online screening programme, by city and year, Hubei Province, China

City	No. of women screened					
	2017	2018	2019	2020	2021^a^	Total
Enshizhou	90 260	51 345	74 485	45 034	NA	261 124
Ezhou	3 913	2 377	7 254	2 849	NA	16 393
Huanggang	69 821	55 882	67 138	45 369	745	238 955
Huangshi	6 219	6 371	12 821	9 163	1 940	36 514
Jingmen	16 326	14 654	20 850	13 274	121	65 225
Jingzhou	24 489	21 943	37 977	21 678	NA	106 087
Qianjiang	2 739	9 179	7 502	5 894	NA	25 314
Shennongjia	79	1 538	1 166	704	902	4 389
Shiyan	39 707	50 310	69 229	33 147	13 328	205 721
Suizhou	11 886	16 576	18 819	13 576	28	60 885
Tianmen	4 109	3 192	9 302	5 414	6 466	28 483
Xiangyang	24 254	20 554	22 006	20 168	7 554	94 536
Xianning	8 746	32 396	41 543	19 266	12 840	114 791
Xiantao	4 689	2 530	8 809	5 368	NA	21 396
Xiaogan	34 030	17 188	36 534	19 974	2 630	110 356
Yichang	28 830	27 040	40 446	23 372	9 115	128 803
**Total**	370 097	333 075	475 881	284 250	55 669	1 518 972

NA: not applicable.

^a ^Cities with NA had met their cytology-based screening coverage targets ahead of schedule.

**Table 2:** Outcomes of the online cervical cancer screening programme, Hubei Province, China, January 2017–December 2021

Outcome	Women screened (*n* = 1 518 972)
**Diagnosis on initial cytology screening, no. (%) **	
Negative for intraepithelial lesion or malignancy	1 388 140 (91.39)
Atypical squamous cells of undetermined significance	60 029 (3.95)
Low grade squamous intraepithelial lesion	21 045 (1.39)
Atypical squamous cells, cannot exclude high-grade squamous intraepithelial lesion	3 401 (0.22)
High-grade squamous intraepithelial lesion	2 148 (0.14)
Atypical glandular cells	25 (< 0.00)
Total positive samples	86 648 (5.70)
Inadequate samples	44 184 (2.91)
**Result of examination of women with positive samples, no. **	
Women who needed a colposcopy-directed biopsy	30 486
**Confirmed diagnosis, no.**	
Women with a colposcopy-directed biopsy	19 495
Women with cervical precancerous lesions	2 785
Women with cervical infiltrating cancer	191

The cost of the screening programme for the public payer was 6.31 United States dollars (US$) per woman, including administration and screening costs (Table 3). The screening fee was paid as a package by the public payer to Landing. The other health service providers involved in the screening also shared the cost based on the actual work done. Most of this fee was intended to cover the various screening services provided by health workers to the women. From the perspective of Landing, the technology provider, the project implementation costs also included training for three types of health worker which is not covered by traditional screening services, including US$ 29.34 per person for the training of 3174 sampling health workers, US$ 293.40 per person for the training of 100 diagnostic health workers and US$ 440.10 per person for the training of 40 cytopathologists. The costs to Landing also included US$ 0.07 per specimen for transport (Table 3).

**Table 3:** Cost of online cervical cancer screening programme, Hubei Province, China

Service or goods	Cost, US$	
	Total for the programme	Per person
**For the public payer**		
Administration^a^	1 564 541.16	1.03^b^
Online screenings by the technology provider^c^	8 020 172.16	5.28^b^
**For the technology provider**		
Training for sampling health workers	93 125.16	29.34^d^
Training for diagnostic health workers	29 340.00	293.40^e^
Training for cytopathologists	17 604.00	440.10^f^
Specimen transport	106 328.04	0.07^b^

US$: United States dollars.

^a^ Includes organization, advocacy and management of digital screening archives.

^b^ Total number of women screened was 1 518 972.

^c^ Includes liquid-based cytology slide preparation, ThinPrep cytological test reports, re-examination and biopsies.

^d^ Total number of trained sampling health workers was 3174.

^e^ Total number of trained diagnostic health workers was 100.

^f^ Total number of trained cytopathologists was 40.

Note: The technology provider was Landing Artificial Intelligence Industry Research Institute, Wuhan, China.

## Discussion

Our online cervical cancer screening programme aided by artificial intelligence was successful in several ways. The low cost, high quality and quick results of the online cytology-based screening were beneficial to large numbers of women. The programme increased the efficiency of data acquisition and tracking in the initial cytology-based screening, reduced reliance on a large number of cytopathologists and had a high participation rate. However, the programme encountered several challenges, such as: the difficulty for some women to travel to the sampling facilities; the inadequate sampling proficiency of sampling health workers at local health centres; the high workload of reviewing negative cases; and the difficulty in specimen conservation and transportation for remote areas with few women. We developed some mitigation strategies that were successfully applied during the programme and have continued until now (Box 2).

Box 2 **Measures used to mitigate challenges to the online cervical cancer screening programme, Hubei Province, China, January 2017–December 2021**

- Establishment of sub-laboratories outside of Wuhan. We reached agreements with some local maternal and child health hospitals to set up laboratories with devices linked to the online screening platform, which allows the hospitals to perform the same procedures as the Landing laboratory in Wuhan.
- Use of portable hand-held automated scanners. The technology provider (Landing Artificial Intelligence Industry Research Institute) developed a hand-held version of the automated scanner. This scanner is less efficient than the laboratory version, but it was used in remote areas where there were few women so that their data could be uploaded to the screening management platform immediately after sampling. This measure allowed these women to be included in the screening, and reduced the cost of sample transportation to Wuhan for diagnosis.
- Deployment of the sampling bus. The Landing Artificial Intelligence Industry Research Institute deployed this bus to provide safe, door-to-door sampling services for women who had difficulty travelling to sampling centres. The bus had the same standardized operating environment as the sampling centres.
- Deployment of trained sampling health workers from local maternal and child hospitals to local health centres. These health workers were sent to assist the sampling health workers at the local health centres who were inexperienced in sampling.
- Deployment of trained diagnostic health workers. The work of the diagnostic health workers was a full-time job that focused on detecting all negative samples using cytological assessments aided by artificial intelligence, and classifying positive cases based on the Bethesda System. The use of these diagnostic health workers led to a substantial reduction in the work of the cytopathologists who would otherwise have had to review the large number of negative samples.

Because of a lack of funding for development, diagnostic facilities and qualified medical staff, local health centres are often underused^31^^,^^32^ and not trusted^33^ in China. However, because they provide long-term routine health care to targeted women in the community or village and thus have a good foundation for interaction with the population, sampling at local health centres as the first stage of the screening process may increase women’s participation in cytology-based screening.^34^ We arranged for local maternal and child health hospitals to assist the less skilled and experienced health workers at local health centres in completing the sampling. This approach allowed local health centres to improve their service skills while also receiving some financial assistance through the public payment channel; it also gave women the opportunity to access screening services near their homes.

**Fig. 2:**
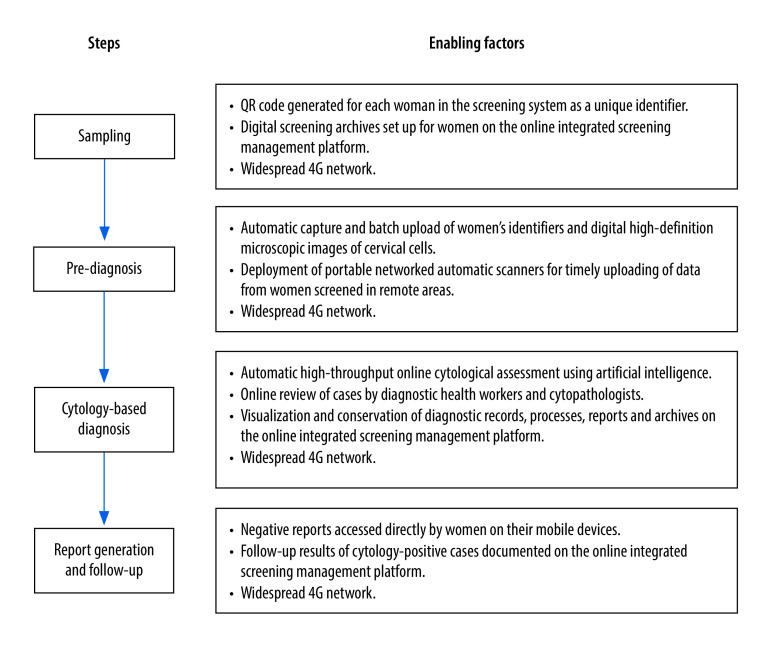
Enabling factors of the online cervical cancer screening programme, Hubei Province, China, January 2017–December 2021

The use of effective cytology assessment algorithms with good performance to replace cytopathologists in mass cytological assessment was an important factor in the success of our programme (Fig. 2). Previous studies have shown that the online cytological assessment system aided by artificial intelligence that we used had a 5.8% better sensitivity than humans in identifying cervical intraepithelial neoplasia grade 2 and above.^27^ A multicentre clinical trial reached a similar conclusion and provided additional findings, for example, that the specificity of artificial intelligence was higher than that of experienced cytopathologists while maintaining the same sensitivity, and that both the sensitivity and specificity of artificial intelligence were higher than with less experienced cytology physicians.^26^ Artificial intelligence-enabled methods are also consistent in quality,^35^ and offer a significant advantage over cytopathologists in traditional screening who need to maintain the quality of their diagnostic skills and avoid errors.^25^^,^^30^ Of the women screened, only 5.70% had positive samples; for cytopathologists to find these positive cases would take more time or would be more labour-intensive. However, in our online cytological assessments, the diagnostic health workers reviewed a sample in an average of 10–12 seconds, which allowed them to evaluate 2500–3000 samples a day.

4G: fourth generation; QR: quick response.

Noteworthy is that online cytological assessment using artificial intelligence can only be used as a reference for diagnosis, and the final diagnostic report must still be reviewed by a cytopathologist. If cytopathologists were still assigned to review the large number of negative cases and potential false positives,^26^^,^^27^^,^^36^ their workload would not be reduced. What we hoped by implementing this type of cytological assessment was that experienced cytopathologists would be able to focus on interpreting both uncomplicated and difficult positive cases that arose from the screening, and that large populations in resource-constrained areas would be able to access good-quality screening services. We achieved this aim by positioning full-time trained diagnostic health workers between the cytological assessment and cytopathologists’ reviews.

If local computing resources are constrained, completing cytological assessments of large populations might be time-consuming. We therefore made the running of the algorithms dependent on a reliable online platform to obtain the required computing power and achieve simultaneous processing of case data.^37^ In Hubei Province, where the 4G network is widespread, pending cases could be uploaded in time for automatic processing as long as the data upload terminals were in a location where they could connect to the network; demands for data downloads could also be met at any time (Fig. 2).

The screening programme had cost advantages for the public payer and the cost per woman was low. Since there are economies of scale associated with this standardized, batched and automated screening service using artificial intelligence, this low cost per woman can be further reduced as the number of women screened continues to increase.^38^

Concerns have been raised that the use of emerging information and communication technologies may not facilitate coverage of new health services, but rather exacerbate health inequalities for vulnerable groups such as elderly people, rural dwellers and those of lower socioeconomic status.^39^^–^^43^ However, we designed our programme to use such technologies to both increase access to screening services for vulnerable groups of women (Fig. 2) and ensure the stability of the programme during the coronavirus disease 2019 (COVID-19) pandemic. Specifically, several types of networked automated scanners provided greater flexibility and speed in collating data on the women and the samples. Since the online platform synchronized data generated and uploaded by any terminal device in the system, we could easily access data on screening progress, diagnostic operations, quality control and screening archives for administrative supervision and evaluation. Most importantly, the use of the online artificial intelligence system, diagnostic health workers and cytopathologists enabled successful, rapid and quality-assured cytological assessments. Even though COVID-19 affected some of the planned work on site, we took steps to mitigate these issues. We used online announcements and short message notifications to encourage women’s participation in the screening, while on-site meetings were mostly postponed. Appointments for sampling and treatment were arranged online to limit the number of women who could attend on-site services each day.

Our study has some limitations. No standard measures were in place for women whose slides were considered inadequate. Some women were called immediately and successfully resampled, while others, especially rural women, were unable to complete the resampling because they were often moving to find work. Some women who were notified were resampled several months later. Inadequate samples are unavoidable because of sampling errors, so it is important that resampling for women with such samples is officially planned.

Women with cervical precancerous lesions and cancer in low- and middle-income countries are often unsupported without timely detection. In accordance with WHO’s goal of eliminating cervical cancer and China’s campaign for universal coverage of cervical cancer screening, we have shown that an online service-delivery system using artificial intelligence can provide increased access to screening for poor, remote and migratory women. The efficient design of such systems requires a comprehensive approach that includes the use of digital technologies, the training and monitoring of health workers, the support of local government agencies and effective plans for implementation of screening.

Future research should focus on: evaluation of the programme’s key technologies and workforce deployment; addressing any problems with this new screening model; and exploration of the potential integration of other services using computer technology.
